# Computational identification of 4-carboxyglutamate sites to supplement physiological studies using deep learning

**DOI:** 10.1038/s41598-021-03895-4

**Published:** 2022-01-07

**Authors:** Sheraz Naseer, Rao Faizan Ali, Suliman Mohamed Fati, Amgad Muneer

**Affiliations:** 1 Department of Computer Science, University of Management and Technology, Lahore, 54770 Pakistan; 2 Computer and Information Sciences Department, Universiti Teknologi PETRONAS, 32610 Seri Iskandar, Malaysia; 3 College of Computer and Information Sciences, Prince Sultan University, Riyadh, 11586 Saudi Arabia

**Keywords:** Biochemistry, Molecular biology, Biomarkers, Diseases, Mathematics and computing

## Abstract

In biological systems, Glutamic acid is a crucial amino acid which is used in protein biosynthesis. Carboxylation of glutamic acid is a significant post-translational modification which plays important role in blood coagulation by activating prothrombin to thrombin. Contrariwise, 4-carboxy-glutamate is also found to be involved in diseases including plaque atherosclerosis, osteoporosis, mineralized heart valves, bone resorption and serves as biomarker for onset of these diseases. Owing to the pathophysiological significance of 4-carboxyglutamate, its identification is important to better understand pathophysiological systems. The wet lab identification of prospective 4-carboxyglutamate sites is costly, laborious and time consuming due to inherent difficulties of in-vivo, ex-vivo and in vitro experiments. To supplement these experiments, we proposed, implemented, and evaluated a different approach to develop 4-carboxyglutamate site predictors using pseudo amino acid compositions (PseAAC) and deep neural networks (DNNs). Our approach does not require any feature extraction and employs deep neural networks to learn feature representation of peptide sequences and performing classification thereof. Proposed approach is validated using standard performance evaluation metrics. Among different deep neural networks, convolutional neural network-based predictor achieved best scores on independent dataset with accuracy of 94.7%, AuC score of 0.91 and F1-score of 0.874 which shows the promise of proposed approach. The iCarboxE-Deep server is deployed at https://share.streamlit.io/sheraz-n/carboxyglutamate/app.py.

## Introduction

Cells, the fundamental units of life, experience different physiological phenomena during their lifecycle which give rise to the dynamic changes in their structure and functions. One such phenomenon is post-translational modification of proteins, the complex molecules, which are found in nearly all aspects of cell’s life^1^. An important post-translational-modification (PTM) is 4-Carboxyglutamate (CarboxE), synthesized by replacing a proton from 4-carbon of glutamate with carboxyl group^2^. 4-Carboxyglutamate plays pivotal role in the blood clotting cascade specifically occurring in Coagulation factors II, VII, IX, and X, protein C, protein S, and some bone proteins^3^. Aforementioned coagulation factors are dependent on Vitamin-K, a cofactor for carboxylase, which serves as a catalyst for CO$_2$ addition on glutamate peptide for carboxylation^4^. Oxygenation of vitamin K hydroquinone is catalyzed by Vitamin K-dependent carboxylase, a bi-functional enzyme, enabling the creation of vitamin K epoxide which forms carboxyglutamate^5^. Furthermore, a key part is played by CarboxE in calcium dependent interaction between prothrombin and negatively charged phospholipid surface, which is pivotal for activation of prothrombin to thrombin^6^. CarboxE is found to have significantly lower mean disorder scores than their unmodified counterparts and the modified residues showed lower mean spatial fluctuations than unmodified residues^7^. The CarboxE containing proteins of hepatic origins are characterized by their role in blood coagulation while non-hepatic proteins containing same PTM, e.g. osteocalcin and atherocalcin, are known for their calcium binding properties^8^. Additionally, the small amounts of osteocalcin and other CarboxE containing proteins are found to occur in calcified atherosclerotic lesions and mineralized heart valves^9^. Due to its calcium binding properties, CarboxE is also considered a biomarker for diseases including osteoporosis, papilloma, bone resorption and plaque atherosclerosis^3,5^.

Owing to the importance of CarboxE in physiological phenomena, research has been done on identification of carboxylation sites using mass spectrometric analysis^10^. But due to huge cost and effort requirements for in-vivo, ex-vivo and in-vitro identification of CarboxE, scarce effort is put in wet lab identification of the same. Meanwhile, in-silico methods, based on machine learning and data science, showed a promising avenue to characterize CarboxE sites to supplement wet lab methods. In fact, researchers have applied in-silico methods to support the wet lab experiments in proteomics and genomics using various machine learning and artificial intelligence techniques^11–17^. Prior literature proposed various computational methods to identify glutamate carboxylation^5,18^. While these contributions show promise, the proposed computational models were based on human-engineered features. According to Lecun et al.^19^, human-engineered features suffer from certain limitations as they are difficult to calculate because of absence of a feedback mechanism between prediction algorithm and feature extraction mechanism. The absence of feedback system hinders the development of an effective predictor because there is no way to evaluate the quality of features beforehand. Additionally, generation of human-engineered features require domain knowledge and human intervention which is costly to achieve^19^.

**Figure 1:**
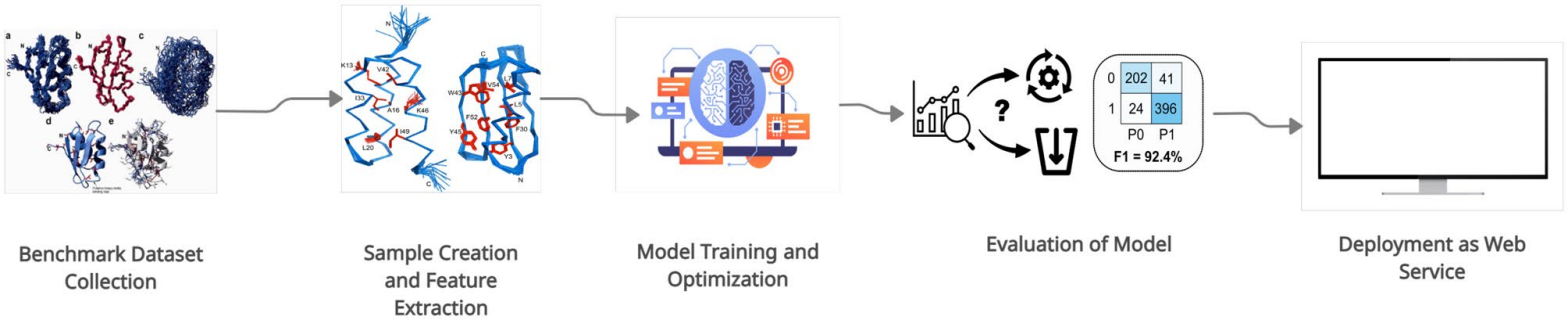
Chou’s five step methodology.

Modern deep learning offers a very powerful framework for solving learning problems. When a Deep Neural Network (DNN) is sufficiently trained on input/output pairs of peptide sequences and labels, it is able to reduce the input sequence, by performing hierarchical input transformations through trained hidden layers of neurons, into the correct label for given input peptide sequence. DNNs do not require prior feature extraction, because the deep model can automatically learn the low-dimensional, task specific and optimal feature representation from hierarchical non-linear transformations of original pseudo Amino ACID Composition (PseAAC) sequences. These abstract, task specific deep neural representations are used by the output layer, which is usually composed of any classifier like sigmoid or softmax, to make predictions^20–22^. In effect, deep learning is gaining popularity for solving the proteomics and genomics problems due to non-requirement of prior costly feature extraction^23–25^. Deep learning provides a highly powerful framework for handling learning challenges in the modern-day. Although, the majority of the works for PTM prediction are comprised of conventional machine-learning-based feature extraction methodology, deep learning is gaining popularity to solve proteomics and genomics problems due to the non-requirement of prior costly feature extraction^14,26,27^. Deep learning based models are far more efficient and provide results comparable conventional Machine-learning predictors as demonstrated in^22,26,27^. Another significant advantage of deep learning is lack of need for Feature-engineering or extraction because DNNs can work with raw inputs. Our methodology uses DNN based approach to identify potential CarboxE sites and will make sure that devised improvements are met in the best possible way. In contrast to previously proposed conventional ML based predictors, which rely on quality of features, the current analysis aims to devise an in-silico approach for CarboxE site prediction by fusing DNNs with Chou’s five-step rule^28^ as presented in Fig. 1 and used extensively by previous studies^5,11–17^.

## Results

**Figure 2:**
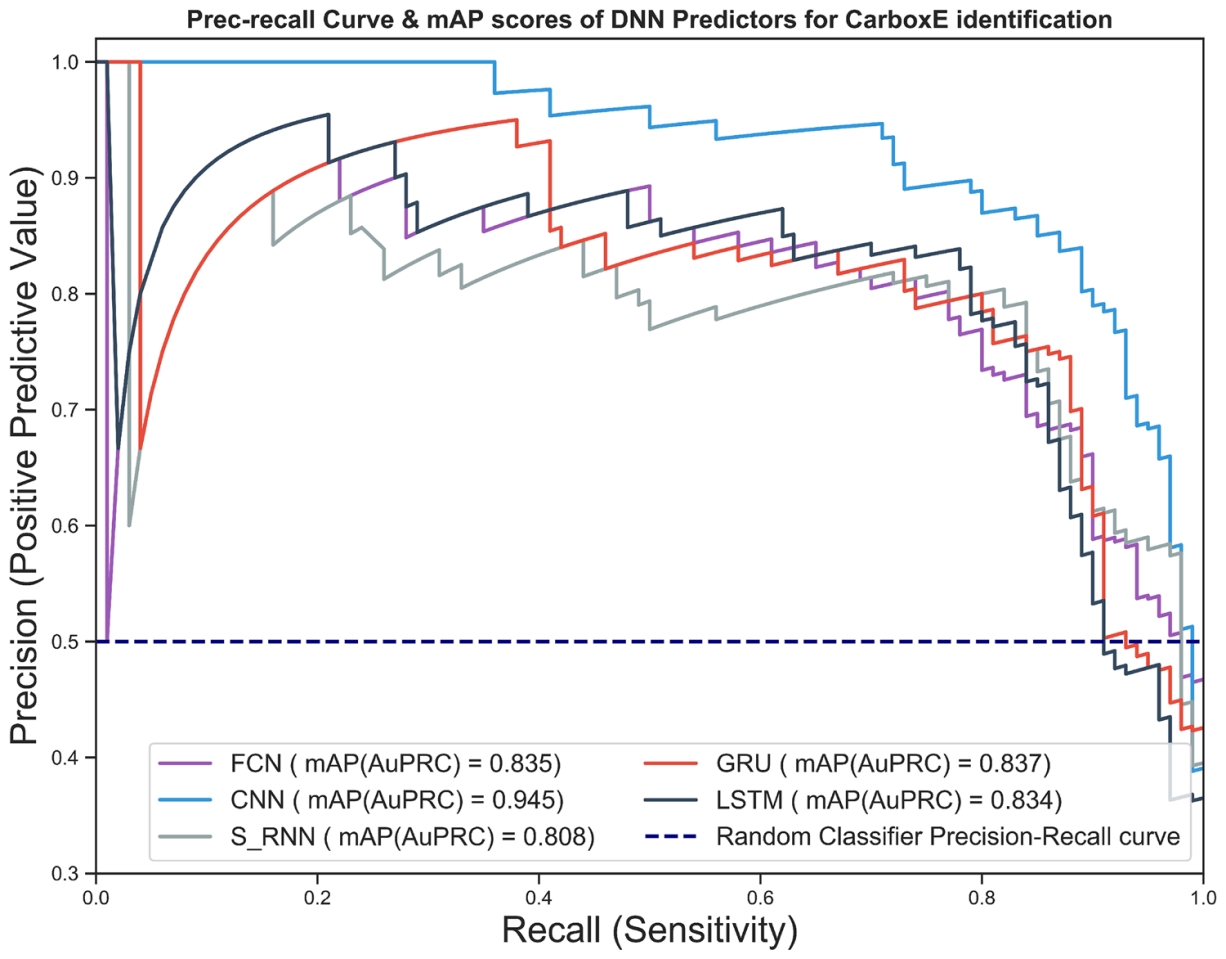
Precision-recall curve and mAP scores DNN-based CarboxE site prediction models.

In this research, DNNs-based model performance is evaluated using well-known evaluation metrics. The critical evaluation metrics employed in this study include the receiver operating characteristics learning curve (ROC), precision-recall, Area under Curve, accuracy, and matthew’s correlation coefficient to name a few. A brief description of above-mentioned metrics is discussed in following section . The five proposed DNNs models are evaluated and tested on the testing data set, which is not exposed to models throughout the training process, to guarantee fair estimation of generalization capability. The following subsections offers evaluation results of DNN based predictors for identifying CarboxE sites developed in this study. Figure 2 illustrates the precision-recall curve of candidate DNN based predictors. As depicted in Fig. 2, the CNN model’s curve is closest in the precision-recall space to the perfect prediction point (1, 1) compared to that of the other classifiers, demonstrating the better performance of the CNN classifier model. In comparison to the aforementioned CNN based model, all the other DNN-based classifiers performed below par, as shown by their respective curves, which are comparatively far from the perfect classification point. To represent the findings of precision-recall curve in a single scalar value, mean average precision (mAP) is used which is defined as the region under the precision-recall curve. The higher the mAP score of the classifier, the greater the classifier’s prediction efficiency and vice versa. For all candidate CarboxE site prediction models, the mean average precision (mAP) scores for DNN models are presented in the legend portion of Fig. 2. As illustrated in aforementioned figure, the CNN-based model achieved the best score of 0.937 and LSTM achieved the best second score with a value of 0.876. Meanwhile, FCN fell short and obtained the lowest score of 0.723. Overall, the DNN models utilized in this study achieved a score higher than 70%.

**Figure 3:**
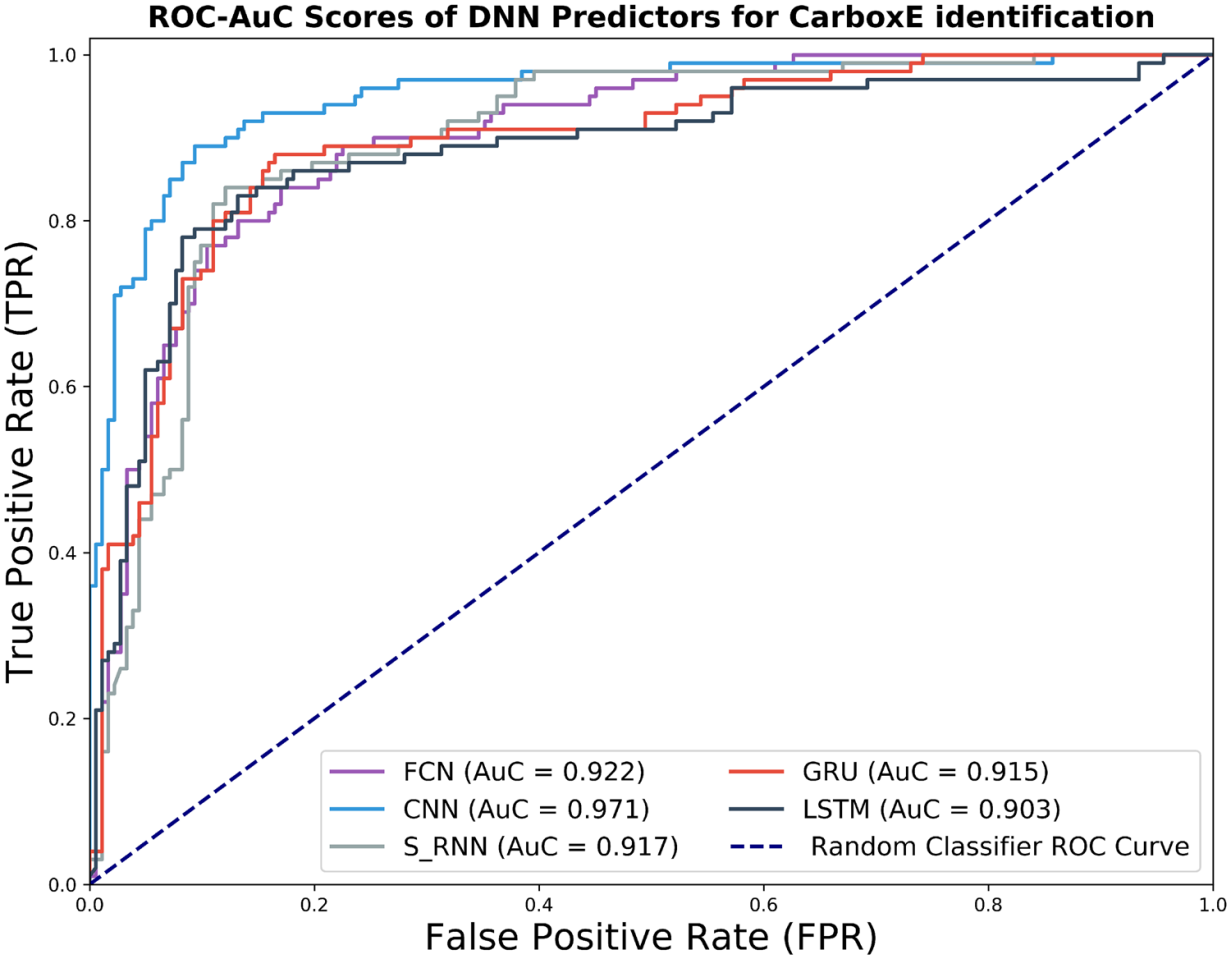
ROC curve and AUC scores for DNN based CarboxE site identification models.

### Receiver operating characteristics and area under ROC curve

The ROC curves for the proposed five DNN based CarboxE predictors, built in this study, are introduced in Fig. 3. It can be seen from the aforementioned figure that the curve of the CNN-based predictor is nearest to the perfect classification point as compared to that of remaining DNN based models, demonstrating the better performance of the CNN-based model. The AUC values for the models built in this analysis are presented in the Legend portion of Fig. 3. It is shown clearly from the aforementioned figure that the CNN based model outperforms the rest of the methods in predicting CarboxE sites, with an AUC value of 0.971. The FCN model obtained the second-best prediction with an AUC value of 0.922. The results of the ROC curve corroborate the earlier evaluation results indicated by the precision-recall curve.

**Figure 4:**
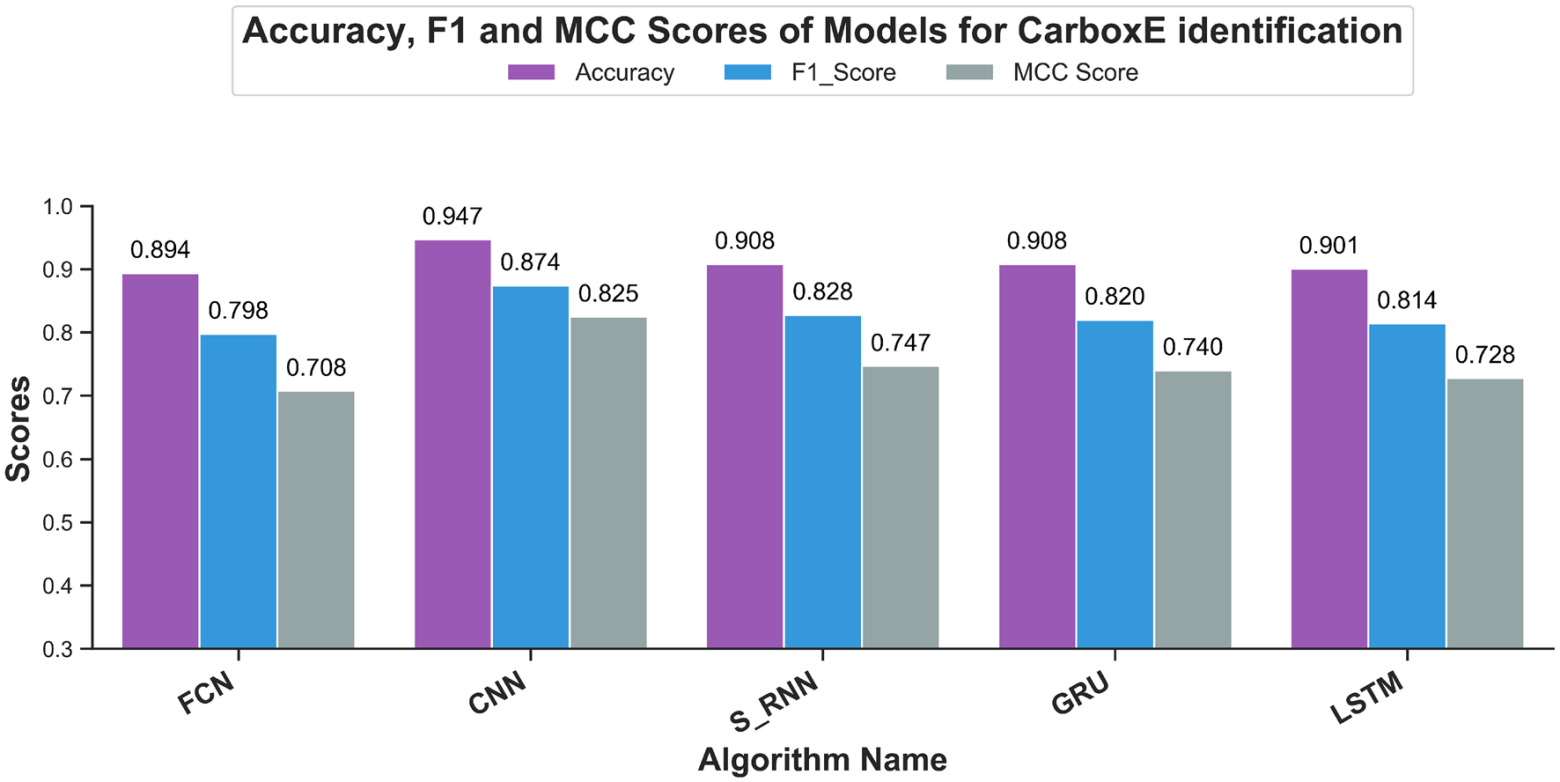
Accuracy, F1-measure, and MCC scores of DNN-based CarboxE site prediction models.

### Accuracy, F1-measure, and Matthew Correlation Coefficient

The accuracy score of CarboxE identification models, calculated using independent testset, are illustrated in Fig. 4. From the aforementioned figure, it is evident that the accuracy score of CNN-based predictor dominated remaining all predictors developed in this study with score of 0.947 followed by 0.908 score of GRU and S-RNN based models. Although the accuracy results are trust-worthy for balanced datasets, it can be misleading when an imblanace exists in data points of different classes in a dataset. To mitigate the possibility of its spurious findings, accuracy is often used in conjunction with F1 score or matthew’s correlation coefficient. The F1-measurements of CarboxE identification models are also depicted in Fig. 4 and the results validate the domineering performance of CNN-based model which showed F1-score of 0.874 while S-RNN model remained the runner-up with F1-measurement of 0.828. GRU and LSTM based models showed comparable performance and achieved an F1 score of 0.820 and 0.814, respectively. FCN score wasn’t that far from the aforementioned DNNs but achieved last place nonetheless with F1-score of 0.798. The outcomes of MCC for all DNN dependent models, proposed in this study, are shown in Fig. 4. Based on MCC, the CNN-based model achieved best performance rate of 0.825, followed by the S-RNN-based and GRU-based models achieving a performance rate of 0.747 and 0.740. Lastly, the FCN-based model obtained the least performance score of 0.708 in terms of MCC evaluation matric. From the performance of CNN based model in all three point metrics discussed in this section, it is evident from the evaluation scores that CNN based model showed promising performance and surpassed other DNN based model developed during this study.

### Comparison with literature

This subsection discusses the comparison of proposed approach with similar contributions from literature as well the prospective reasons for better performance of DNNs for identification of CarboxE sites. Identification of CarboxE using machine learning and other in-silico methods is relatively ignored area of research by bioinformatics community and we were able to find only one contribution for in-silico prediction of 4-carboxyglutamate by Shah et al.^5^. Shah et al. used statistical moments and residue position related techniques to extract features form the peptide sequences and used these features to train their 4-carboxyglutamate predictor. The comparison of our best model i.e. CNN based model with system proposed by^5^ is presented in Table 1 on the basis of standard performance evaluation metrics. It can be seen from Table 1 that the model proposed by Ref.^5^ is performing almost as good as the proposed predictor. The reader may feel that if the predictor developed by Ref.^5^ is performing comparable to proposed approach then there is little merit in using the DNNs for the problem at hand. This is not the case owing to the following benefits provided by proposed approach:

- Human engineered feature extraction is generally more expensive and requires human experts to develop and validate the features. The system proposed by Ref.^5^ uses statistical moments and position incidence which are costly to extract, needs expert domain knowledge and human intervention to achieve better results^5^.
- Deep features, as used in current study, are more advantageous than human engineered features because they do not require any human intervention and are easily extractable, once the deep model is trained. The deep features can be extracted efficiently by a forward pass through the trained DNN.
- Deep features are usually simpler and more effective than their human engineered counterparts because, in DNNs, the feature extraction and classification work in unison to extract the features which help to achieve better features for classification. This is evident form fact that the proposed CNN based approach, in this study, uses 8 deep features (extracted from last fully connected layer of CNN) to achieve comparable classification results to the system proposed by Shah et al.^5^ which uses more than 100 human engineered features to train classifiers.

Aforementioned facts illustrate the merits of DNN based prediction of CarboxE sites and compel us to consider DNN based model an effective and efficient alternative for rather expensive approaches utilizing human engineered features^22^.

**Table 1:** Comparison with available literature.

Performance evaluation metric	Proposed CNN based model	Reported results by^5^
Accuracy	0.947	0.94
AUC	0.971	0.96
F1-score	0.874	Not reported
MCC	0.825	0.85
Sensitivity	0.923	0.92
Specificity	0.918	0.93
mAP	0.945	Not reported

### Model deployment as webserver

**Figure 5:**
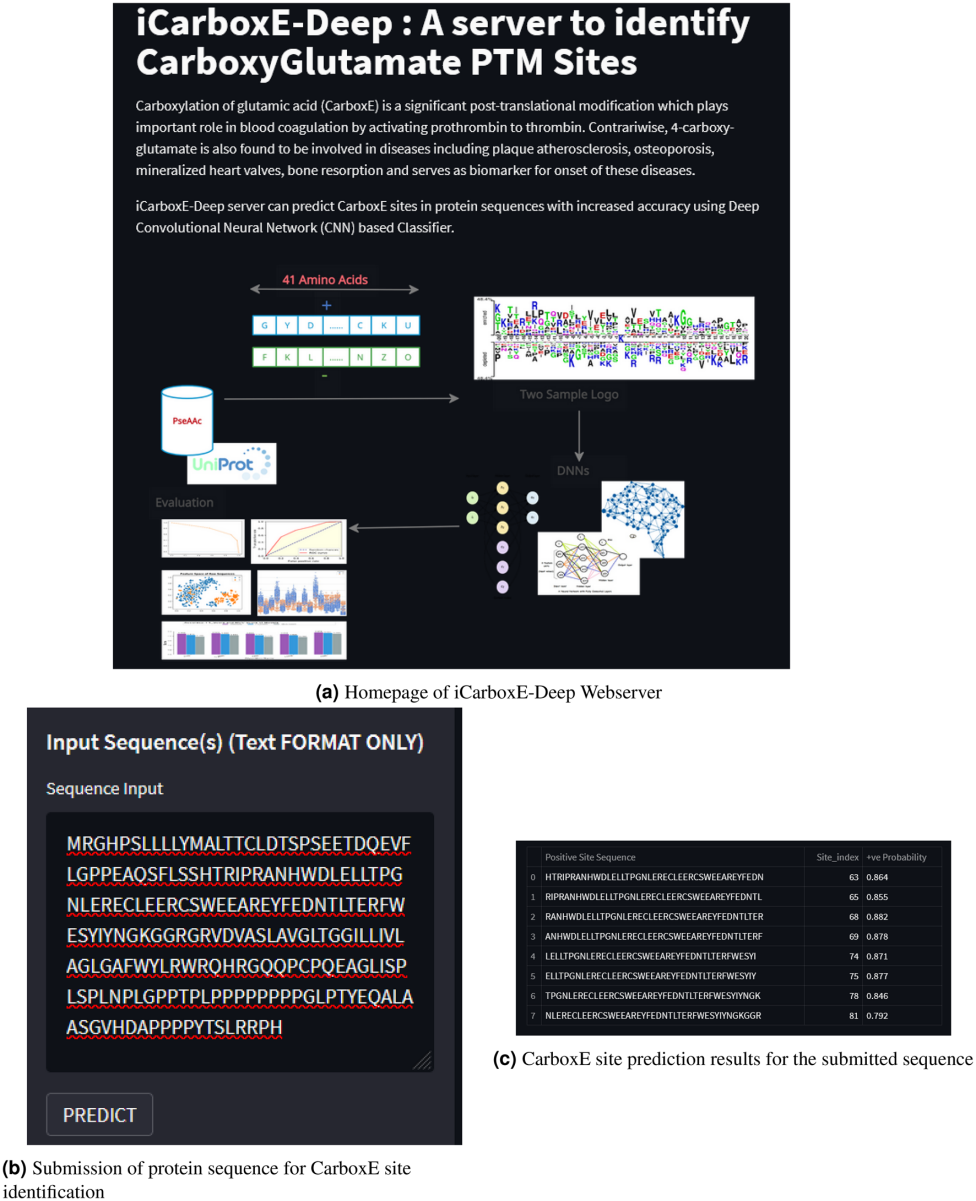
iCarboxE-Deep Webserver functionalities for identification of 4-carboxy-glutamate.

Final step of Chou’s 5-step rule as shown in Fig. 1 is the deployment of developed model as a web service to enable easy access for research community. To this end, we developed a web application based on our best performing CNN based model for identification of CarboxE sites. The webserver is temporarily deployed at https://share.streamlit.io/sheraz-n/carboxyglutamate/app.py. The web application can accept a peptide sample in the form of string and return the identified glutamic acid sites likely to be carboxylated. Homepage of iCarboxE-Deep webserver is shown in Fig. 5a while Fig. 5b highlights the peptide sequence submission process for computing CarboxE sites. Figure 5c illustrates result page showing the identified glutamic acid sites likely to be carboxylated and the corresponding $\xi = 41$ length PseAAC sequence of residues.

## Discussion

**Figure 6:**
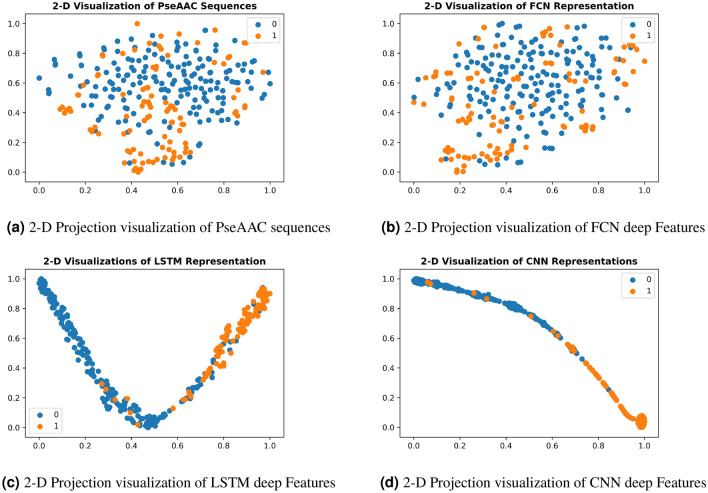
Feature space visualizations of deep representations for positive and negative CarboxE sample.

**Figure 7:**
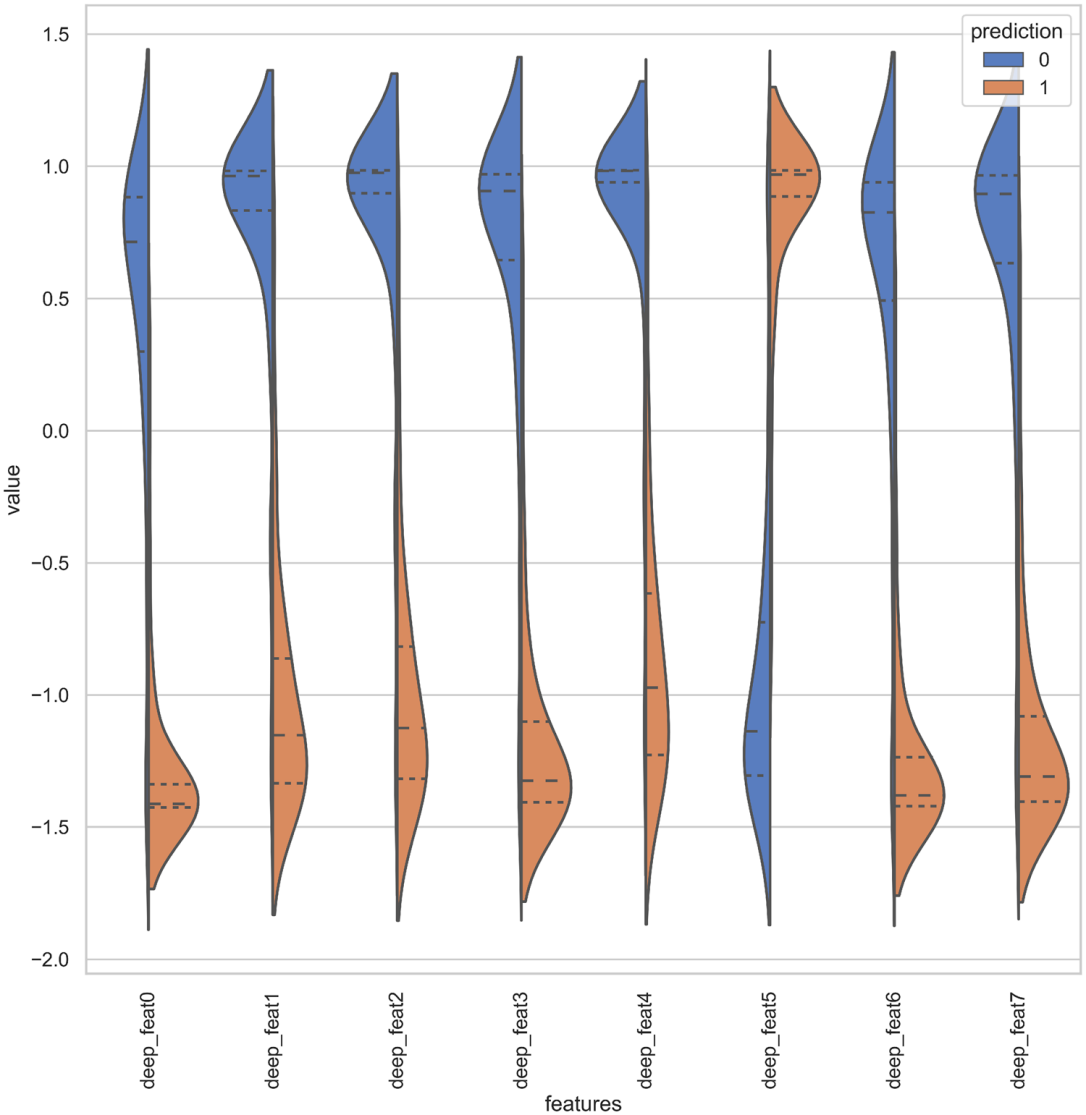
Violin plot of positive and negative class distributions learned by CNN representation.

For understanding deep feature representations of peptide sequences, learned by DNNs to predict CarboxE sites, visualizing these feature spaces can provide an intuitive understanding of why these feature representations work. To create these visualizations, we calculated the output of penultimate layer of each trained model using testset peptide sequences and extrapolated the 2-D projections of the same using t-stochastic neighborhood embeddings (t-SNE) algorithm, developed by Maaten and Hinton^29^. T-SNE makes use of non-linear statistical approach to extrapolate 2-D projections of deep features calculated from non-linearly transformed input peptide sequences. T-SNE uses many hyperparameters including perplexity, initialization and iterations to develop the projections in lower dimensions. Since our testset contained only 308 samples with maximum 41 dimensions for raw sequences and 8 dimensions for deep representations, the recommended range for perplexity is 0–50. We used default perplexity value of 30 for scikit-learn t-SNE implementation^30^, used PCA initialization for efficient dimensionality reduction and fixed the iterations to 1000 for calculating the 2-D projections of deep features. The developed 2-D projections of deep models were plotted on the basis of class labels using matplotlib and seaborn package of python. Fig. 6a–d show the aforementioned visualizations of PseAAC sequences and feature space representations learned by the deep models developed in this study. Visualization of Raw PseAAC sequences, as visible in Fig. 6a, shows the distribution of positive and negative CarboxE samples without any feature extraction. As illustrated in the figure, the samples from both classes are cluttered over the space and no clear boundary exists between samples of two classes. This chaotic distribution suggests that any classifier aiming to separate samples of both classes while using this representation will have a hard time doing so. Figure 6b–d depict the effect of non-linear transformations of three DNNs, used in this study, to separate both classes in respective feature space for achieving better predictions. The visualization plots included in manuscript corresponds to one low performance model i.e FCN and two optimal models including LSTM and CNN based models. The FCN feature space visualization is shown in Fig. 6b. It can be verified from aforementioned figure that this model was not sufficiently successful in separating the positive and negative samples before passing their representation to output layer which resulted in poor performance of respective predictor. The best class separation is achieved by the input representation learned by CNN model as shown in Fig. 11. The data distribution of positive and negative samples in CNN representation is illustrated in violin plot shown by Fig. 7. It is evident from Figs. 6d and 7 that this representation is not chaotic and cluttered and both classes are sufficiently separated to make the job of classifier comparatively easier. This means any classifier consuming this representation to predict CarboxE sites will be able to distinguish between both classes with less effort and achieve better predictions. This is also corroborated by the better results shown by CNN based predictor as discussed in “Results”.

The major benefit of DNN based approach proposed in this study is the automatic feature representation learning using stochastic gradient decent. Proposed approach removes the requirement to use costly feature engineering process. Moreover, the proposed DNN based predictor of this study are only the first step towards employing deep learning for 4-Carboxyglutamate site identification and research community can extend this study to come up with more effective in-silico systems using deep learning for 4-Carboxyglutamate site identification.

**Figure 8:**
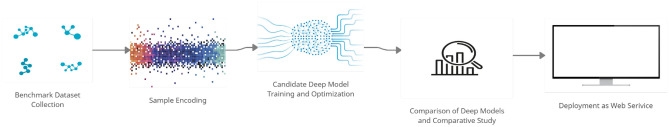
Proposed approach for Carboxyglutate(CarboxE) sites identification.

## Materials and methods

The suggested approach for this study, as shown in Fig. 8, is derived from the five-step rule of Chou^28^, popular in proteomics research^31,32^. However, instead of depending on human-engineered features, the proposed approach employs DNNs for combining feature extraction and model training to extract features and train models and use the intrinsic capabilities of DNN’s feature extraction and classification. If the DNN model is satisfactorily trained, the hidden layers of DNN perform processing on PseAAC peptide sequences to calculate effective deep representations, which are then utilized by the DNN’s output layer to perform prediction. The loss score is used as a feedback signal by the optimizer to enhance both the feature extraction and classification capability of the model. In this study, several DNN-based models have been trained and tested to arrive at an optimal model for predicting CarboxE sites. This section’s key purpose is to elaborate the first three phases presented in Fig. 8, while rest have been explained in previous sections.

### Benchmark dataset collection

**Figure 9:**
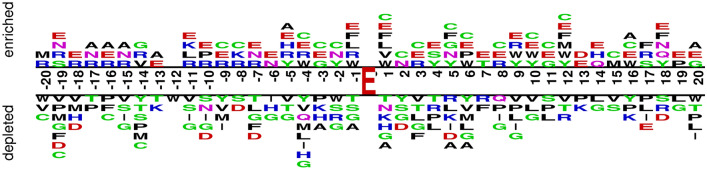
Two sample logo of Benchmark Dataset.

We utilized the advanced search and annotation features in UniProt^33^ to produce a dataset for conducting the proposed study. The benchmark dataset’s consistency has been ensured by choosing protein sequences that are experimentally investigated and evaluated. Selected proteins were subjected through CD-Hit^34^ to remove the homology with a threshold of 0.8. Resulting proteins were used to extract positive and negative sequences for CarboxE sites. The PseAAC representation of a peptide sequence containing a positive CarboxE site may be described as follows:

$$\begin{aligned} f_{\epsilon }(P) = k_{-\epsilon } k_{-(\epsilon -1)} \dots k_{-2} k_{-1} \mathbf{E } k_{+1} k_{+2}\dots k_{+(\epsilon -1)}, k_{+\epsilon } \end{aligned}$$

where ‘E’ denotes PTM site for CarboxE and ‘k’ represents the neighbor amino acid residues of positive site. Respectively, the Greek letter $\epsilon$ describes the indexes of PseAAC sequence residues, where the left-hand residues of CarboxE site are located at negative $\epsilon$ indexes, and the right-hand residues are located at their respective positive $\epsilon$ indexes. To develop a benchmark dataset, the length $\xi$ for both negative and positive samples were extracted from experimentally verified proteins. Based on empirical observations and literature support^5,26,27^, the length $\xi$ is set at 41 for negative and positive samples equally. Each positive sample is created via setting the index of the CarboxE site at 21 and collecting 20 left and 20 right neighbor residues of the positive side, which resulted in the standard $\xi$ length sequence. For sequences with $\xi < 41$, a dummy residue symbol ‘X’ is placed on both sequence sides to obtain the standard length. Similar approach was utilized to develop the negative samples from aforementioned experimentally verified proteins, where the only difference is the presence of non-CarboxE glutamate at sequence index $\epsilon =21$ rather than CarboxE site. Using the above process, we were able to get 308 positive and 617 negative samples. The final benchmark dataset comprised of 308 positive and randomly chosen 617 negative samples making a total of 925 samples. The final dataset can be represented as follows:

$$\begin{aligned} E= E^{+} \cup E^{-}, \end{aligned}$$

where $E^{-}$ denotes negative 617, and $E^{+}$ denotes positive, 308 samples. Class proportions of the positive and negative reference groups were 33% and 67%, respectively. The benchmark dataset of this study is available at https://mega.nz/folder/NgcSXLzY#CaBCn-f4190fgO_Qj4iNpQ. Authors in Ref.^35^ have suggested two-sample logo that is created to visualize residues that are substantially depleted/enriched in the collection of CarboxE fragments to help develop understanding about sequence biases around CarboxE sites. As shown in Fig. 9, the benchmark dataset two-sample logo comprises forty-one residues, twenty upstream and twenty downstream, from all Glutamate (CarboxE and non-CarboxE) sites present in experimentally validated CarboxE proteins. The positive sample contains 338 samples consisting of experimentally confirmed CarboxE sites, while the negative sample contained remaining non-redundant Glutamate sites from same group making a sum of 925. There were significant differences in the enriched region (containing CarboxE sites) and depleted region (containing non-CarboxE sites). P, G, and V were more frequently observed in the depleted position, while E, C, and R were more regularly noticed in the enriched region. Multiple amino acid residues were discovered stacked at certain over-or under-represented positions in the neighboring sequences, meaning that there is a substantial difference between the positive and negative samples. The findings show that more task-specific and non-linear features are needed to differentiate between both groups of samples.

### Sample encoding

DNNs require input sequences in the form of quantitative data to process. A simple quantitative encoding of the PseAAC sequences was utilized to minimize the encoding technique’s impact, as presented in Table 2. Quantitative encoding is done according to Table 2, where the first row shows IUPAC amino acid symbols and the corresponding integer in the second row defines the encoding used for the sample. A useful outcome of this encoding technique is the minimal effect of encoding on the final results. The benchmark dataset has been divided into a training set of 647 samples, and a testing set of 278 samples with a ratio of 70/30. However, both training and testing sets maintained the original class ratio.

**Table 2:** Amino acid encoding utilized in this research.

X	A	C	D	E	F	G	H	I	K	L	M	N	O	P	Q	R	S	T	U	V	W	Y
0	1	2	3	4	5	6	7	8	9	10	11	12	13	14	15	16	17	18	19	20	21	22

### Candidate model training and optimization

This section focuses on describing the DNNs architecture and optimization utilized to develop CarboxE site prediction candidate models. This study has employed commonly used neural network architectures like “Standard Neural Networks (FCNs), Convolutional Neural Networks (CNNs), and Recurrent Neural Networks (RNNs) with simple units, Gated Recurrent Unit (GRU) and Long Short-Term Memory (LSTM) units, respectively. For DNN optimization, we applied the Randomized Hyperparameter search methodology employed in Ref.^36^ to maximize the effectiveness of DNN candidate models. A randomized search over large hyperparameter space presents better hyperparameters for DNNs with a finite number of computations. In this strategy, Hyperparameters are randomly sampled, and models created using these parameters are evaluated. The following subsections present a quick overview of each DNN architecture that is utilized to predict the CarboxE sites.

#### Standard neural network

A standard neural network (FCN) is composed of layers of neurons in a manner that each neuron in the previous layer is associated with all neurons in the following layer. The FCN is aimed to estimate the learning function $f^{*}$ where $f^{*}$ is a classifier described as $y=f^{*} (\alpha ,x)$ and use appropriate parameters $\alpha$ to assign appropriate category label y to input x. The FCNs’ task is to discover the optimal set of parameters $\alpha$ so the $y=f^{*} (\alpha ,x)$ mapping provides the best possible approximation to $f^{*}$.

**Figure 10:**
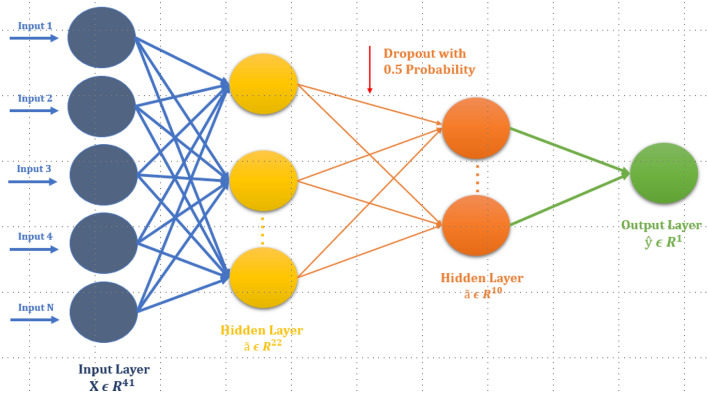
Architecture of FCN for CarboxE site identification.

To predict CarboxE sites, an FCN architecture comprising of three dense layers of 38, 18 and 8 rectified linear neurons (relu) respectively is used, as shown in Table 3, along with a dropout layer to minimize over-fitting. A single Sigmoid neuron served as the output layer for the binary classification task. The FCN architecture is illustrated in Fig. 10. Stochastic gradient descent (SGD) optimizer is used to train the model, with a learning rate of 0.01 via minimization of negative logarithmic loss. The training set was further divided into a training set and a validation set with a ratio of 70/30 for FCN based CarboxE predictor training. It is important to note that the test set, to evaluate the resulting CarboxE site prediction models’ generalization capability, was never shown during the training phase to FCN and other DNNs. After the model was successfully trained, the evaluation was done using the benchmark test set, and the performance was assessed by utilizing well-known measurement metrics.

**Table 3:** Standard neural net architecture for identification of CarboxE site.

No	Layer	No. of weights
1	Dense layer with 22 relu units	$(41 + 1) \times 22 = 924$
2	Dropout with 0.5 probability for Regularization	No weights
2	Dense layer with 10 relu units	$(22 + 1) \times 10 = 230$
3	Output layer with single Sigmoid unit	$(10 + 1) \times 1 = 11$

#### Recurrent neural networks

**Figure 11:**
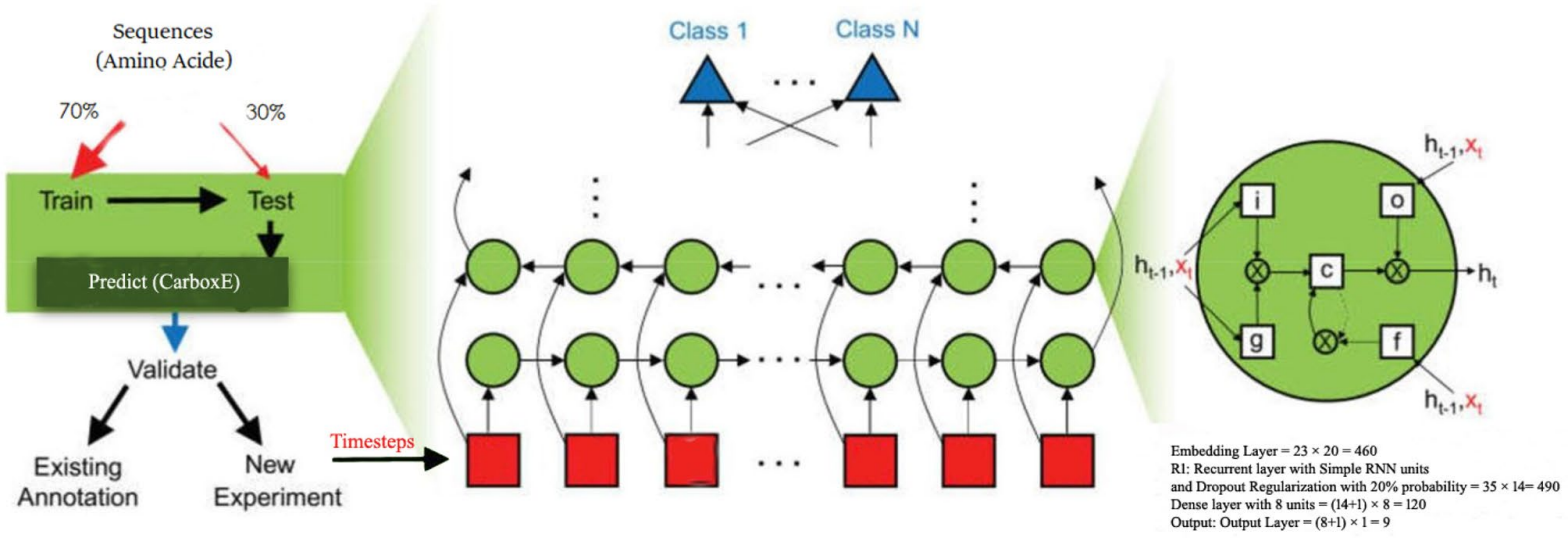
Architecture shared by RNNs to identify CarboxE sites.

A shortcoming of traditional DNNs is that the weights are learned by individual neurons which preclude the DNNs from identifying exact representations that occurred at different locations in sequences. An RNN circumvents the restriction via utilizing a repeating loop over timesteps to resolve the problem mentioned above. A sequence vector ${x_{1},\dots ,x_{n}}$ is manipulated utilizing a recurrence of the form $a_{t}=f_{\alpha } (\gamma _{t-1},x_{t})$, where learning function is denoted by f, $\alpha$ is a set of parameters applied at each time step t and $x_{t}$ is the input at timestep t. Three variations of recurrent neurons i.e., a simple RNN unit, a gated recurring unit (GRU), and the LSTM unit are used to develop the candidate RNN based models for the proposed study. The shared architecture of three RNNs is shown in Fig. 11 where the green circles of RNN show recurrent cells while red squares show timesteps i.e. residue vectors of peptide sequence being classified by the model. At each timestep in a simple recurrent neuron, the weights governing the connections from the input to the hidden layer, between previous activation $a^{t-1}$ & current activation $a^{t}$, and from the hidden layer to the output layer, are shared. A basic recurrent neuron’s forward pass can be expressed as follows:

$$\begin{aligned} \begin{aligned} a^{t}&= g(W_{a}[a^{t-1}, X^{t}]+ b_{a}) \\ y^{t}&= f(W_{y} \times a^{t} + b_{y}), \end{aligned} \end{aligned}$$

where g reflects an activation function, ‘t’ represents the current timestep, $X^{t}$ outlines input at timestep t, $b_{a}$ defines the bias, $W_{a}$ presents cumulative weights and the activation output of timestep t is denoted by $a^{t}$. If needed, this $a^{t}$ activation could be employed to measure the $y_{t}$ forecasts at time t. Table 4 demonstrates the RNN method structural design with the simple RNN neurons. This model uses an embedding layer to predict the amino acid sequence in vector space $R^{20}$, and transform the semantic relationships into geometric relationships. The following layers of the DNN model interpret these sequence vectors’ geometric relationships to learn deep feature representations, which are evaluated by the output layer to render predictions. To make predictions output layer is developed using a single sigmoid unit. Even Though DNNs with simple RNN neurons enjoy favorable outcomes in several applications, they remain susceptible to vanishing gradients and demonstrate a limited capability to learn long-term dependencies. The research community has provided several modified recurrent neuron architectures to overcome the simple RNN neurons drawback. Well-known architectures include the Gated Recurrent Unit (GRU) technique proposed by Ref.^37^ and the LSTM method presented by Ref.^38^ to resolve the problem of gradients disappearing and to allow long-term dependences to be learned. Cho et al.^37^ presented GRU, which is capable of showing better performance for long-term relationship learning in sequence data. The memory variable $H^{t}$, which contains the running summary of samples seen by the neuron till timestep t and is given by $H^{t}= a^{t}$ is used by the GRU unit at each stage t, which provides an updated list of the entire samples processed by the unit. Hence, the GRU unit considers overwriting the $H^{t}$ at each timestep t, but the regulation of memory variable overwriting is implemented via the update gate $\Gamma _{u}$, when the GRU unit superimposes the $H^{t}$ value at each step ‘t’ with the candidate value ${\bar{H}}^{t}$. GRU neuron functionality can be represented via the following series of equations:

$$\begin{aligned} {\bar{H}}^{t}&= tanh(W_{c}[\Gamma _{r} \times H^{t},X^{t}]+b_{c} \\ \Gamma _{r}&=\sigma (W_{r}[H^{t-1},X^{t}]+b_{r}) \\ \Gamma _{u}&= \sigma (W_{u}[H^{t-1},X^{t}]+b_{u}) \\ H^{t}&= \Gamma _{u} \times {\bar{H}}^{t}+(1-\Gamma _{u}) \times H^{t-1} \\ a^{t}&= H^{t}, \\ \end{aligned}$$

where $W_{r}$, $W_{c}$ and $W_{u}$ represents the respective weights and $b_{r}$, $b_{c}$ and $b_{u}$ denote the subsequent bias terms for input $X_{t}$ at timestep t. $\sigma$ is the function of logistic regression, and the activation value at timestep t is represented by $a^{t}$. Except for the usage of GRU neurons, the implemented RNN model developed with GRU is like that of simple RNNs. Table 5 presents the GRU-based RNN model architecture for CarboxE site identification.

**Table 4:** RNN architecture using SimpleRNN neurons for CarboxE site identification.

No	Layer	No. of weights
1	Embedding layer	$(2320 = 460)$
2	R1: Recurrent layer with 14 simple RNN units and dropout regularization with 20% probability	$(3514= 490)$
3	Dense layer with 8 units	$(14+1)8 = 120$
4	Output layer	$(8+1)1 = 9$

**Table 5:** CarboxE site identification using RNN based on GRU neurons.

Layer type	No. of weights
Embedding layer to convert numeric sequence into vector sequence	$(23 \times 20 = 460)$
Recurrent layer with GRU units and dropout regularization with 20% probability	$(108 \times 14 = 1512)$
Dense layer with 8 units	$(14+1) \times 8 = 120$
Output layer	$(8+1) \times 1 = 9$

**Table 6:** CarboxE site identification using RNN based on LSTM neurons.

Layer type	No. of weights
Embedding layer to convert numeric sequence into vector sequence	$(23 \times 20 = 460)$
Recurrent layer with LSTM units and dropout regularization with 20% probability	$144 \times 14 = 2016$
Dense layer with 8 units	$(14+1) \times 8 = 120$
Output layer	$(8+1) \times 1 = 9$

As mentioned earlier, Hochreiter et al.^38^ have proposed the LSTM neuron with some improvements to the design of the SimpleRNN unit, which provides a more robust generalization of GRU. Prominent variations in LSTM and GRU cells are illustrated as follows:

- No significance gate $\Gamma _(r)$ is used in generic LSTM units for ${\bar{H}}^{t}$ computation.
- LSTM units utilize two distinct gates instead of an update gate $\Gamma _{u}$, namely output gate $\Gamma _{o}$ and update gate $\Gamma _{u}$. The output gate tracks the content’s visibility of the $H^{t}$ memory cell to compute LSTM unit activation outputs for other hidden units in the network. To achieve $H^{t}$, forget gate handles the extent of overwriting on $H^{t-1}$. For instance, how much memory cell information must be overlooked to function properly for memory cells?
- LSTM is different from GRU architectures by the fact that the memory cell contents $H^{t}$ may not be equivalent to the activation $a^{t}$ at time t.

Moreover, the Model using RNN-LSTM approach is constructed with similar architecture as GRU and simple RNN models. The only difference is that of LSTM units in recurrent layers. Table 6 shows the model’s architecture that used LSTM neurons and RNNs to build the CarboxE site identification model.

#### Convolutional neural networks

**Figure 12:**
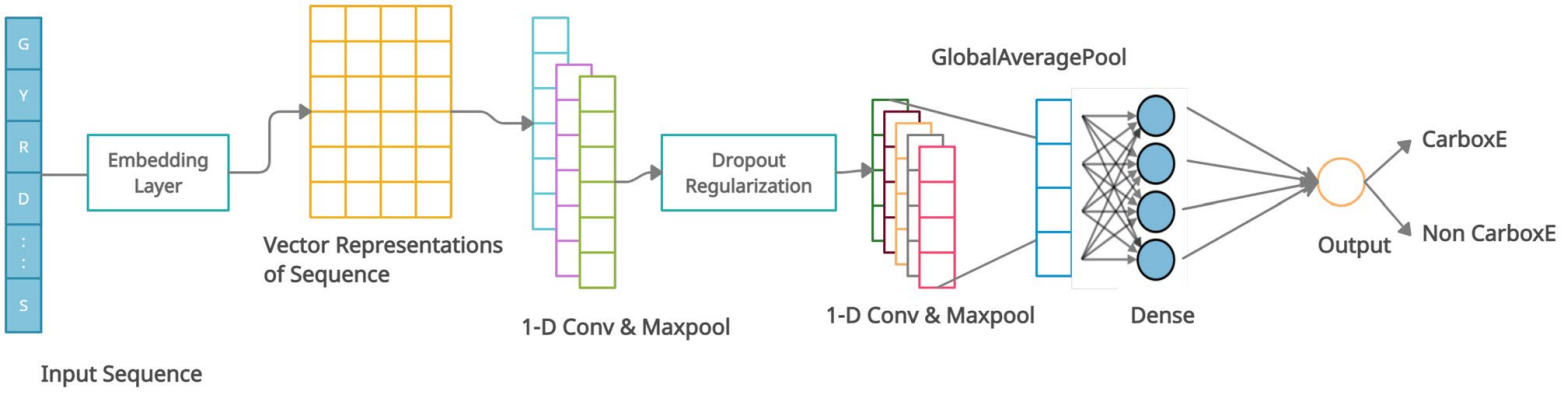
CNN architecture to identify CarboxE sites.

Convolutional Neural networks are designed to handle learning problems involving large input data with complex spatial structures such as image, video, and speech signals. CNNs try to learn hierarchical filters which can transform large input data to accurate class labels using minimal trainable parameters. This is accomplished by enabling sparse interactions between input data and trainable parameters through parameter sharing to learn equivariant representations (also called feature maps) of the complex and spatially structured input information^20^. In a Deep CNN, units in the deeper layers may indirectly interact with large portion of input due to usage of pooling operations which replaces the output of Net at a certain location with a summary statistic and allows the network to learn complex features from this compressed representation^20^. The so-called ’top’ of the CNN is usually composed of a bunch of fully connected layers, including the output layer, which uses the complex features, leaned by previous layers, to make predictions. The CNN-based architecture of the CarboxE site identification approach is shown in Fig. 12. CNN model for CarboxE identification makes use of an embedding layer, two convolution-maxpool blocks separated by a Dropout layer, a global average layer, penultimate feature extraction layer and an output layer consisting of the sigmoid neuron as shown in Table 7. Each peptide sample x with a length $\xi = 41$ was translated via the embedding layer to achieve $X \in R(\eta \times \xi )$tensor where $\eta \in R^{20}$ is the symbol vector in $R^{20}$ of every amino acid residue. The first conv-maxpool block is comprised of 8 1-D convolution neurons with a filter size of 3 with relu non-linearity followed by a 1-D maxpool operation. The second conv-maxpool is similar in architecture, with the only difference being the increased number of neurons to 18. Two Dropout layers, proposed by Srivastava et al.^39^, are employed to reduce the overfitting during the training phase. The GlobalAveragePooling layer flattens the output of previous layers in a one-dimensional array of 18 values by calculating an average of each of the 18 feature maps of previous layers. The 18-D feature array is used by ‘top’ of the CNN, consisting of fully connected layers of relu, to identify CarboxE sites.

**Table 7:** CarboxE site identification model based on CNN.

Layer type	No. of weights
Embedding layer to convert numeric sequence into vector sequence	$(23 \times 20 = 460)$
Conv-maxpool-1D block with 10 filters of size 5	$((5 \times 20) + 1) \times 10 = 1010$
Dropout with 25% of probability	N/A
Conv-maxpool-1D block with 16 filters of size 3	$((3 \times 10) + 1) \times 16) = 496$
GlobalAveragePooling1D	N/A
Dropout with 50% of probability	N/A
Dense layer with 8 units	$(16+1) \times 8 = 136$
Output layer	$(8+1) \times 1 = 9$

### Evaluation methodology

The critical evaluation metrics employed in this study include the receiver operating characteristics learning curve (ROC), precision-recall, Area under Curve, accuracy, and matthew’s correlation coefficient to name a few. All the above-mentioned metrics stem from the confusion matrix, which is composed of the following measures:

- True Positive (TP): Actual CarboxE site forecasted via DNN classifier as CarboxE site
- False Positive (FP): Actual non-CarboxE site indicated via DNN classifier as CarboxE site
- False Negative (FN): Actual CarboxE site indicated via DNN classifier as non-CarboxE site
- True Negative (TN): Actual Non-CarboxE site forecasted via DNN classifier as non-CarboxE site

This subsection provides a brief introduction of the evaluation metrics for convenience of interested readers.

#### Precision-recall curve and mean average precision

When considering the identification models’ evaluation, recall and precision are considered crucial measures. Recall evaluates the classifier’s sensitivity to positive samples and is depicted by the ratio of correct positive predictions and total positive samples in the test. At the same time, precision evaluates the relevance of the predicted positive samples and is calculated as the ratio of correct positive predictions to total positive predictions. A high precision and recall ranking indicate that the predictions made via model for the positive class contain a high percentage of true positives (high-Precision), together with identification of majority of positive class samples in the dataset (High-Recall). A precision-recall curve is determined by plotting precision and recalls against each other, and it evaluates the proportion of positive identifications that are true positives^40^. In precision-recall space, the closer a predictor’s score is to the ideal classifier point (1, 1) the better it is and contrariwise.

#### Receiver operating characteristics and area under ROC curve

A receiver operating characteristics (ROC) is a method for organizing, visualizing, and selecting classification models based on their performance^41^. Additionally, it is a valuable performance evaluation measure as it is insensitive to changes in class distribution and especially useful for problems involving skewed class distributions^41^. The ROC curve illuminates, in a sense, the cost-benefit analysis under evaluation of the classifier. The false positive (FP) ratio to total negative samples is defined as the false positive (FP) rate and measures the negative examples misclassified fraction as positive. This is considered a cost since any further action taken on the FP’s result is considered a waste, as it is a wrong prediction. True positive rate, defined as the fraction of correctly predicted positive samples, can be considered an advantage due to the fact that correctly predicted positive samples assist in solving the problem being examined more effectively. RoC curve is created by plotting the False Positive Rate with True Positive Rate. In ROC space, point (0, 1) represents the perfect classifier because this point depicts FPR of 0 with TPR of 1. The closer a curve is to this ideal point, the better the performance and contrariwise. Additionally, the ROC curve can be represented as a scalar value using Area under ROC curve (AUC). The AUC is the indicator of a classifier’s capability to differentiate between classes, and it is employed as an ROC curve summary. AUC reduces the effects of the ROC curve to a single value and highlights mathematical insights into the success of the model. AUC is equal to the probability that a randomly chosen positive sample will be classified higher than a randomly chosen negative instance by the classifier. Moreover, AUC is similar to the Wilcoxon rank test^41^. The greater the AUC score, the better the model distinguishes the negative and positive samples^42^ and vice versa.

#### Accuracy, F1-measure, and Matthew Correlation Coefficient

Accuracy is defined as the ratio of correctly estimated data points to the total number of data points and its a widely accepted evaluation measure for classification models. Although its results are trust-worthy for balanced datasets, it can be misleading when their exist an imblanace in data points of different classes in a dataset. To mitigate the possibility of its spurious findings, accuracy is often used in conjunction with F1 score or matthew’s correlation coefficient. F1-score may be understood as an average of precision and recall and it is used when a scalar representation of aformenetioned measures is desired. Thus, the F1 score can be defined as given in equation below:

1 $$\begin{aligned} F1 = 2 \times \frac{precision \times recall}{precision+recall}. \end{aligned}$$

Another noteworthy point metric is Matthews Correlation Coefficient (MCC)^42,43^, which was initially proposed to compare chemical structures^44^ but found its use as standard performance metric for classification models^45^. MCC has been shown to be robust agianst class imbalannce issues which are prevalent in other model evaluation measures. The MCC is a more robust statistical metric that produces a high score only if classifier obtained good results for all four confusion matrix measures (true positives, false negatives, true negatives, and false positives) proportionate to both positive and negative class size in the test dataset.

## Conclusions

In this study, we proposed an efficient in-silico approach to supplement wet lab experiments for identification of 4-carboxyglutamate sites. 4-carboxyglutmate is an important post translational modification which is involved in various physiological processes including blood coagulation and pathological conditions like osteoporosis etc. The proposed approach employs Chou’s Pseudo Amino Acid Composition with deep neural networks to identify glutamic acid sites likely to be carboxylated. Well-known deep neural networks including standard neural network, three RNNs with different neuron structures and convolutional neural network were used to develop identification models for 4-carboxyglutamate sites. Of all DNN based predictors, highest position was surmounted by CNN based model, which showed the best results on independent dataset with accuracy of 94.7%, AuC score of 0.91 and F1-score of 0.874. The comparisons of proposed CNN based predictor with notable research contributions were performed which shows the efficacy of proposed predictor. On the basis of abovementioned evidence, it is concluded that the proposed CNN based predictor will help the research community to efficiently and accurately identify 4-carboxyglutamate sites and help develop better understanding of related pathophysiological processes.
